# The Triglyceride to HDL Ratio and Its Relationship to Insulin Resistance in Pre- and Postpubertal Children: Observation from the Wausau SCHOOL Project

**DOI:** 10.1155/2012/794252

**Published:** 2012-06-28

**Authors:** Karen Olson, Bryan Hendricks, David K. Murdock

**Affiliations:** ^1^Aspirus Heart and Vascular Institute-Research and Education, Wansau, WI 54401, USA; ^2^University of Wisconsin-Madison, Madison, WI 53706, USA

## Abstract

Insulin resistance (IR) is a risk factor for ischemic heart disease and diabetes and raises the triglyceride/high-density lipoprotein (TG/HDL) ratio in adults, but is not well defined in children. *Purpose*. To investigate the TG/HDL ratios in children as an IR marker. *Methods*. Wausau SCHOOL Project assessed 99 prepubertal and 118 postpubertal children. The TG/HDL ratio was correlated with numerous risk factors. *Results*. TG/HDL ratio was significantly correlated with QUICKI, HOMA-IR, zBMI, waist-to hip ratio, systolic and diastolic BP, LDL size and LDL number. A group of 32 IR children (HOMA-IR > 1 SD from the mean, i.e., >2.45) had significantly higher TG/HDL (3.11 ± 1.77) compared to non-IR children (1.86 ± 0.75). A TG/HDL ratio of ≥2.0 identified 32 of the 40 children deemed IR by HOMA-IR (>2.45) with a sensitivity of 0.80 and a specificity of 0.66. Children with TG/HDL ratio ≥3 were heavier and had higher BP, glucose, HOMA-IR, LDL number, and lower HDL level, QUICKI, and LDL size, regardless of pubertal status. *Conclusion*. The TG/HDL ratio is strongly associated with IR in children, and with higher BMI, waist hip ratio, BP, and more athrogenic lipid profile.

## 1. Introduction

Insulin resistance (IR) is a major risk factor for ischemic heart disease and diabetes [1–4]. IR significantly impacts lipoprotein metabolism and is associated with an increase in triglyceride (TG) levels, depressed high-density lipoprotein (HDL) levels, and an increase in the number of small dense LDL particles [4, 5]. The effect of IR on TG and HDL has made the TG/HDL ratio a useful marker of insulin resistance in adults [6, 7]. IR is increasingly identified in children [8]. Much less information is available regarding the impact of IR on lipid metabolism in children. The usefulness of the TG/HDL ratio in children as a marker of IR has not been adequately explored.

The Wausau SCHOOL Project is the result of a community-based effort to investigate the prevalence and magnitude of cardiovascular risk factors in school age children in the Wausau School District [9, 10]. The Wausau SCHOOL Project database includes anthropometric measurements, fasting lipid profiles, and insulin and glucose levels from pre-and postpubertal children. The purpose of this investigation was to use this database to assess the impact of IR on lipid metabolism and determine the correlation of the TG/HDL ratio to IR and other markers of IR.

## 2. Methods

Details of the data collection for the Wausau SCHOOL Project have been described previously [9, 10]. Briefly, the investigation was reviewed and approved by the Aspirus Hospital Institutional Review Committee. A representative sample of Wausau School District students enrolled in grades 2, 5, 8, and 11 were recruited from all 17 schools. Approximately 480 students from each grade were invited to participate [9]. Consent was obtained from parent or guardian as well as assent from participating children. Weight and height measurements, waist and hip circumference, and blood pressure measurements were obtained for each participant. Waist and hip circumference measurements were used to calculate the waist-to-hip ratio [9, 10]. A student was classified as having an elevated blood pressure if the systolic pressure was greater than 120 mm/Hg or the diastolic was greater than 80 mm/Hg on more than one occasion [9]. 

Twelve-hour fasting lipid levels were measured using nuclear magnetic resonance technology (LipoScience). This technique provides a standard lipid profile, as well as a measure of the size and number of low-density lipoprotein (LDL) and high-density lipoprotein (HDL) particles. From the fasting lipid profile, a TG/HDL ratio was calculated for each student. 

The fasting blood sample was also used to obtain glucose and insulin levels. Insulin levels were measured by LipoScience using a commercial chemiluminescent immunometric assay (IMMulite 2000). From the fasting insulin and glucose levels, the HOMA-IR [11] and the QUICKI [12] measurement of IR was calculated for each student. A student was considered IR if the HOMA-IR or QUICKI exceeded 1 standard deviation above the mean [10]. Because puberty affects IR [10, 13], calculations of HOMA-IR and QUICKI were analyzed separately for children in 2nd grade (prepubertal) and 11th grade (postpubertal) [10]. 

The BMI was corrected for age and sex based on revised growth charts developed by the Centers for Disease Control and Prevention (CDC) [14]. Using these data, a standardized body mass index (zBMI) was calculated as a measure of how much each student deviated from the CDC mean. For example, a zBMI of 1 indicates that the student exceeded the mean BMI for a child of that age and sex by 1 standard deviation (SD), while negative numbers indicate values below the mean. Students were considered overweight if they exceeded the 85th percentile in this distribution and obese if they exceeded the 95th percentile [15].

## 3. Statistical Methods

Means and SDs were calculated for the continuous variable data, and the groups were compared by independent group *t*-tests or analysis of variance tests (ANOVAs). Categorical comparisons were made using Chi-square analyses. From the calculations, the TG/HDL ratio was correlated with both the QUICKI and HOMA-IR method of determining insulin resistance, zBMI, and low-density lipoprotein (LDL) particle size and number. Data analyses were conducted using the Statistical Package for the Social Sciences, version 17.0, and *P* < 0.05 was considered significant.

## 4. Results

Of the total 1920 students randomly recruited from grades 2, 5, 8, and 11, 715 were selected to participate in the SCHOOL Project. Of these, insulin, glucose, and lipid levels were obtained in 137 2nd graders and 126 11th graders. This study is based on 234 students who had complete data available, including HOMA-IR and lipid values. The mean HOMA-IR (1.22 ± 1.07) and QUICKI (0.39 ± 0.050) in 2nd graders was significantly different, *t*(167) = 3.71, *P* < 0.001, and *t*(174) = 6.85, *P* < 0.001, respectively, than 11th graders' HOMA-IR (2.18 ± 2.65) and QUICKI (0.36 ± 0.030) and consistent with the postpubertal state being more IR. This higher degree of IR was also reflected in a higher TG/HDL ratio in 11th graders (2.59 ± 1.81) compared to 2nd graders (1.84 ± 1.67), *t*(231) = 3.28, *P* = 0.001. When combining all students, the TG/HDL ratio was significantly correlated with both the HOMA: IR [*r*(232) = 0.319, *P* < 0.001] and the QUICKI [*r*(233) = −0.403, *P* < 0.001] method of assessing IR. 

Table 1 summarizes correlations among TG/HDL and IR as measured by HOMA-IR and QUICKI, as well as to the lipid measurements, and anthropometric measurements. The TG/HDL ratio was significantly correlated with all of these parameters. Thus a higher TG/HDL ratio was associated with heavier students who were more likely to have smaller and more numerous LDL particles.

A TG/HDL of greater than 2.0 identified 32 of the 40 children deemed IR by HOMA-IR (>2.45) with a sensitivity of 0.80 and a specificity of 0.66. A TG/HDL of ≥3.0 was especially specific for IR, with a specificity of 0.90 by the HOMA-IR method and  .72 by the QUICKI method.  Table 2 outlines the characteristic of the students whose TG/HDL level was ≥3 and compares them to the group as a whole. Note the striking differences and high prevalence of characteristics usually associated with IR. Although a TG/HDL ≥ 3.0 was highly specific for IR, the sensitivity for IR as measured by HOMA-IR was reduced to 0.42.

Just as a higher TG//HDL ratio predicted more IR, the converse was also true. The forty children considered IR by the HOMA-IR method had a TG/HDL of 4.05 ± 3.24 compared to a ratio of 1.86 ± .94 in non-IR children (*t*(40) = 4.25), *P* < 0.001. This relationship was true regardless of puberty status. 

## 5. Discussion

We have previously demonstrated that the HOMA-IR and the QUICKI method of assessing IR strongly correlated with zBMI, waist-hip ratios, and lipid abnormalities in pre- and postpubertal children in the Wausau School District [9, 10]. Although HOMA-IR and QUICKI are useful for research purposes, they are expensive and are not readily available to practicing clinician. In the present study, we found that the TG/HDL ratio in children, as in adults, correlates strongly with IR as measured by these two different methods. This relationship was already manifest as early as 2nd grade. Additionally, we found the TG/HDL ratio correlates strongly with other lipid abnormalities commonly found in the IR state: smaller and more numerous LDL particles. Not surprising, this ratio did not correlate as strongly with either total or LDL cholesterol. The poor correlation between IR and measurements of total and LDL cholesterol is well known [5]. Finally, a higher TG/HDL ratio was also strongly correlated with heavier students and a higher prevalence of elevated blood pressure. Thus the TG/HDL ratio in children could be useful in assessing relative IR and help direct resources appropriately to high risk children.

In an attempt to better define the meaning of any given TG/HDL ratio, we calculated the sensitively and specificity of identifying IR students as based on HOMA:IR ≥ 1SD above the mean [10] for a TG/HDL ratio ≥2 and ≥3. Although a ratio ≥2 was quite sensitive for IR (80%), the relatively low specificity made this value less useful. However, a level ≥3 was especially specific for IR. Furthermore, such a level defined a cohort of students also possessing a high incidence of the features commonly associated with IR, including small dense LDL particles, increased BMI, and elevated blood pressure (Table 2). Thus a TG/HDL ratio ≥3 is especially concerning.

The correlation of the TG/HDL ratio in obese children (BMI averaging more than 2 SD above the mean) with IR has previously been made using the glucose clamp, HOMA-IR, and glucose tolerance testing by Giannini et al. [16]. These authors found that the TG/HDL ratio increased as IR increased. Our results confirm and extend upon these findings by including obese and nonobese subjects as well as data on LDL particle size and number and pubertal state.

Bonito et al. [17] also found that the TG/HDL was correlated with IR in children as measured by the HOMA-IR method. Furthermore, they noted that as the TG/HDL ratio increased, so did the incidence of elevated systolic blood pressure and prevalence of increased left ventricular wall thickness on echocardiography suggesting the ratio was a marker of end organ damage. Our results confirm their observations as we also found the TG/HDL ratio to be associated with blood pressure. This was especially evident in those children with a TG/HDL ratio ≥3 (Table 2). 

## 6. Limitation

Because we confined our measurements to pre- and postpubertal states, the meaning of ratios in peripubertal states remains undefined. Additionally, the extrapolation of our findings of students in the Wausau School District to children at large may not be appropriate. Additional investigations of other populations should be performed.

## 7. Conclusion

The results of this study indicate the TG/HDL ratio is strongly associated with IR in children as measured by HOMA-IR and the QUICKI method. An elevated ratio is characterized by a more atherogenic lipid profile, higher waist-hip ratio, and higher BMI. This pattern is already evident by 2nd grade, and the pattern becomes more prevalent in older children. A TG/HDL ≥3 is highly specific for IR and consequently, very concerning. 

## Figures and Tables

**Table 1:** The correlation between TG/HDL ratio and other parameters of insulin resistance.

	TG	TC	LDL	HDL	LDL #	LDL Size	HOMA-IR	QUICKI	WH ratio	zBMI
TC	0.33^∗∗∗^									
LDL	0.20^∗∗^	0.94^∗∗∗^								
HDL	−0.27^∗∗∗^	0.28^∗∗∗^	0.06							
LDL #	0.36^∗∗∗^	0.82^∗∗∗^	0.90^∗∗∗^	−0.19^∗∗^						
LDL Size	−0.36^∗∗∗^	0.24^∗∗∗^	−0.18^∗∗^	−0.48^∗∗∗^	−0.18^∗∗^					
HOMA-IR	0.27^∗∗∗^	−0.02	0.01	−0.28^∗∗∗^	0.09	−0.24^∗∗∗^				
QUICKI	−0.38^∗∗∗^	−0.01	−0.04	0.37^∗∗∗^	−0.13	0.29^∗∗∗^	−0.73^∗∗∗^			
WH ratio	0.16^∗^	0.12	0.11	−0.08	0.13	0.02	0.09	0.03		
zBMI	0.24^∗∗∗^	0.08	0.07	−0.17^∗^	0.14^∗^	−0.16^∗^	0.25^∗∗∗^	−0.34^∗∗∗^	0.06	
TG/HDL	0.92^∗∗∗^	0.18^∗∗^	0.13^∗^	−0.50^∗∗∗^	0.36^∗∗∗^	−0.54^∗∗∗^	0.32^∗∗∗^	−0.40^∗∗^	0.17^∗^	0.26^∗∗∗^

TG: triglyceride, TC: total cholesterol, LDL: low-density lipoprotein, HDL: high-density lipoprotein, LDL #: no. of LDL particles, LDL Size: Size of LDL particles, HOMA-IR: homeostasis model assessment of insulin resistance, QUICKI: quantitative insulin sensitivity check index, WH: waist hip, zBMI: age- and sex- corrected body mass index, TG/HDL: ratio of TG to HDL.

^∗^ *P* < 0.05, ^∗∗^ *P* < 0.01, ^∗∗∗^ *P* < 0.001.

**Table 2:** Clinical characteristics: TG/HDL ratio <3 compared to ≥3.

Characteristic	TG/HDL ratio <3	TG/HDL ratio ≥3	*P* value
TC (mg/dl)	173.0** **±** **28.6	180.2** **±** **38.5	*P * ** **=** **0.294
LDL (mg/dl)	115.4** **±** **25.6	120.0** **±** **32.2	*P * ** **=** **0.352
HDL (mg/dl)	48.3** **±** **9.2	36.7** **±** **7.0	*P * ** **<** **0.001
LDL # (nmol/L)	1147.3** **±** **238.0	1353.9** **±** **388.2	*P * ** **=** **0.004
LDL Size (nm)	21.5** **±** **.36	20.9** **±** **.56	*P * ** **<** **0.001
HOMA-IR	1.46** **±** **1.29	3.29** **±** **4.3	*P * ** **=** **0.017
QUICKI	.38** **±** **.04	.34** **±** **.03	*P * ** **<** **0.001
WH ratio	.84** **±** **.10	.86** **±** **.09	*P * ** **=** **0.269
zBMI	.22** **±** **1.39	1.03** **±** **1.05	*P * ** **=** **0.001
BP-systolic (mmHg)	105.0** **±** **14.9	117.2** **±** **15.4	*P * ** **<** **0.001
BP-diastolic (mmHg)	65.9** **±** **9.8	70.2** **±** **8.2	*P * ** **=** **0.014
Glucose (mg/dl)	83.7** **±** **6.2	86.0** **±** **7.0	*P * ** **=** **0.050
% with elevated BP	34 (17%)	15 (43%)	*P * ** **=** **0.001

TG: triglyceride, TC: total cholesterol, LDL: low-density lipoprotein, HDL: high-density lipoprotein, LDL #: no. of LDL particles, LDL Size: Size of LDL particles, HOMA-IR: homeostasis model assessment of insulin resistance, QUICKI: quantitative insulin sensitivity check index, WH: waist hip, zBMI: age- and sex- corrected body mass index, TG/HDL: ratio of TG to HDL, BP-systolic: systolic blood pressure, BP-diastolic: diastolic blood pressure.

^∗^All *P* values are based on independent *t*-tests, except the last, which is based on a Chi-square with 1** **df. Elevated BP is defined as systolic >120 or diastolic >80.
